# What defines an adaptive radiation? Macroevolutionary diversification dynamics of an exceptionally species-rich continental lizard radiation

**DOI:** 10.1186/s12862-015-0435-9

**Published:** 2015-08-07

**Authors:** Daniel Pincheira-Donoso, Lilly P. Harvey, Marcello Ruta

**Affiliations:** Laboratory of Evolutionary Ecology of Adaptations, School of Life Sciences, University of Lincoln, Brayford Campus, Lincoln, LN6 7DL UK; Laboratory of Evolutionary Palaeobiology, School of Life Sciences, University of Lincoln, Brayford Campus, Lincoln, LN6 7DL UK

## Abstract

**Background:**

Adaptive radiation theory posits that ecological opportunity promotes rapid proliferation of phylogenetic and ecological diversity. Given that adaptive radiation proceeds via occupation of available niche space in newly accessed ecological zones, theory predicts that: (*i*) evolutionary diversification follows an ‘early-burst’ process, i.e., it accelerates early in the history of a clade (when available niche space facilitates speciation), and subsequently slows down as niche space becomes saturated by new species; and (*ii*) phylogenetic branching is accompanied by diversification of ecologically relevant phenotypic traits among newly evolving species. Here, we employ macroevolutionary phylogenetic model-selection analyses to address these two predictions about evolutionary diversification using one of the most exceptionally species-rich and ecologically diverse lineages of living vertebrates, the South American lizard genus *Liolaemus*.

**Results:**

Our phylogenetic analyses lend support to a density-dependent lineage diversification model. However, the lineage through-time diversification curve does not provide strong support for an early burst. In contrast, the evolution of phenotypic (body size) relative disparity is high, significantly different from a Brownian model during approximately the last 5 million years of *Liolaemus* evolution. Model-fitting analyses also reject the ‘early-burst’ model of phenotypic evolution, and instead favour stabilizing selection (Ornstein-Uhlenbeck, with three peaks identified) as the best model for body size diversification. Finally, diversification rates tend to increase with smaller body size.

**Conclusions:**

*Liolaemus* have diversified under a density-dependent process with slightly pronounced apparent episodic pulses of lineage accumulation, which are compatible with the expected episodic ecological opportunity created by gradual uplifts of the Andes over the last ~25My. We argue that ecological opportunity can be strong and a crucial driver of adaptive radiations in continents, but may emerge less frequently (compared to islands) when major events (e.g., climatic, geographic) significantly modify environments. In contrast, body size diversification conforms to an Ornstein-Uhlenbeck model with multiple trait optima. Despite this asymmetric diversification between both lineages and phenotype, links are expected to exist between the two processes, as shown by our trait-dependent analyses of diversification. We finally suggest that the definition of adaptive radiation should not be conditioned by the existence of early-bursts of diversification, and should instead be generalized to lineages in which species and ecological diversity have evolved from a single ancestor.

**Electronic supplementary material:**

The online version of this article (doi:10.1186/s12862-015-0435-9) contains supplementary material, which is available to authorized users.

## Background

Adaptive radiation theory predicts that the proliferation of phylogenetic and ecological diversity within a lineage results from the exposition of a single ancestor to multiple episodes of divergent natural selection [1, 2]. A fundamental component of this process is the emergence of ‘ecological opportunity’, which provides the conditions that allow speciation through adaptation to different niches [3, 4]. Ecological opportunity arises when spatial and/or ecological dispersal (i.e., access to novel niche dimensions facilitated by adaptive innovations) expose a species to a new set of abundant ecological resources [2–7]. For example, spatial and/or ecological dispersal can be driven by the emergence of new habitats (e.g., islands, mountains), by modifications of existing environments via climatic changes, or by the emptying of niches following extinctions [1–3]. As diversification proceeds, the extent of ecological opportunity declines as a function of increasing saturation of niche space by newly evolving species. Therefore, a core prediction based on the above scenario is that adaptively radiating lineages will show early bursts of rapid diversification followed by asymptotic decreases in diversification rates over time [2, 8–10].

In addition, phenotypic traits with ecological significance play a fundamental role in the process of niche construction, and hence, in the way diversifying lineages saturate niches over time [2, 11]. As a result, analyses of macroevolutionary models of lineage accumulation have been complemented with studies of tempo and mode of diversification of ecologically relevant phenotypes during adaptive radiations [2, 8, 12, 13]. Based on the model of adaptively radiating lineages expounded above, we may predict that phenotypic diversification is high early in a group’s history, when ancestors enter an adaptive zone with abundant resources [3, 10]. As natural selection promotes saturation of ecological space via phenotypic diversification, opportunities for niche occupation decline, thus causing a slowdown in the rates of diversification of ecologically functional traits [2, 8–10]. Consequently, if the radiation of a lineage has been adaptive, then the diversifications of both the lineage and the phenotype are expected to display similar patterns, which would be driven by changes in niche filling over time (e.g., [2, 14]). For instance, if the rapid early emergence of new species causes a decrease in niche space, then the opportunities for adaptive speciation decline, and slowdowns in ecological trait evolution would be expected given the reduced opportunities for adaptive niche expansions.

Evidence for coupled patterns of lineage and phenotype diversification is not consistent. While some studies reveal a link between these two components of diversity, others fail to identify such links. For example, Harmon et al. [12] showed that ‘bursts’ of lineage accumulation in the radiation of iguanian lizards are consistent with pulses of phenotypic disparity during their phylogenetic history. Similarly, the radiation of Caribbean *Anolis* lizards has been shown to partition ecological morphospace more finely as the numbers of competing lineages present on an island increase [15]. In contrast, the radiation of cetaceans shows signals of diversity-dependent evolution of ecological phenotypes, while their net diversification fails to support a model of early-bursts of speciation followed by slowdowns [13]. Finally, although net lineage diversification has been rapid and described by a diversity-dependent trajectory in the exceptionally explosive radiation of *Rattus* rats, the extent of interspecific morphological diversification has been minimal [16].

A number of hypotheses have been formulated to explain such disjoint patterns between lineage and phenotype diversity. For example, it has been suggested that the signatures of early burst adaptive radiations can be retained in phenotypic traits, while high extinction or fluctuations in net diversifications can erase them from the structure of the phylogeny [13, 17]. Also, non-adaptive radiations are expected to diversify taxonomically but not much phenotypically [16, 18–20]. Finally, a longstanding debate focuses on whether macroevolutionary processes differ between island and continental radiations. Given that islands are spatially limited and have simpler ecological backgrounds compared to continents, both diversification processes and cladogenesis-phenotype links may follow different trajectories mediated by their intrinsic differences in ecological opportunity, which is expected to be more common on islands [1, 21–23]. In fact, although most biodiversity resides on continents [24], current knowledge on adaptive radiations derives primarily from island models. Therefore, studies of diversification dynamics in both lineages and phenotypes in continental radiations remain both a critical empirical and conceptual need and a promising research venue.

In recent years, the exceptionally diverse radiation of South American lizards of the genus *Liolaemus* has emerged as a promising model to investigate adaptiveradiations on continents. Consisting of 240+ species, *Liolaemus* is the world’s second richest genus of extant amniotes [25]. Remarkably, since their origin (estimated ~22 Mya, [25, 26]), these lizards have adapted to the widest range of ecological and climatic conditions known among reptiles [6, 25, 27, 28], including extreme environments ranging from the Atacama Desert (the driest place on Earth) to Tierra del Fuego (the southernmost place where a reptile has been found), along both the Pacific and Atlantic coasts, and reaching up to 5,000 + m altitudes in the Andes [27, 29–34]. Importantly, recent studies suggest that this radiation may have been accelerated by the enormous ecological opportunity created by the Andes uplift [6, 35]. This idea also suggests that the evolution of viviparity (live-bearing reproduction) provided the key innovation that unlocked the harsh Andean environments for early *Liolaemus* colonisation and subsequent diversification [6, 35, 36]. Thus, this lineage offers a unique model to investigate the causes and trajectories of evolutionary radiations on continents. Here, we study the tempo and mode of macroevolutionary diversification in lineage richness and body size in the *Liolaemus* radiation, and discuss our findings in the context of radiations triggered by continental ecological opportunity. A central prediction derived from adaptive radiation theory is that both diversity dimensions will show signals of diversity-dependent diversification over time.

## Methods

### Phylogenetic tree

**Fig. 1:**
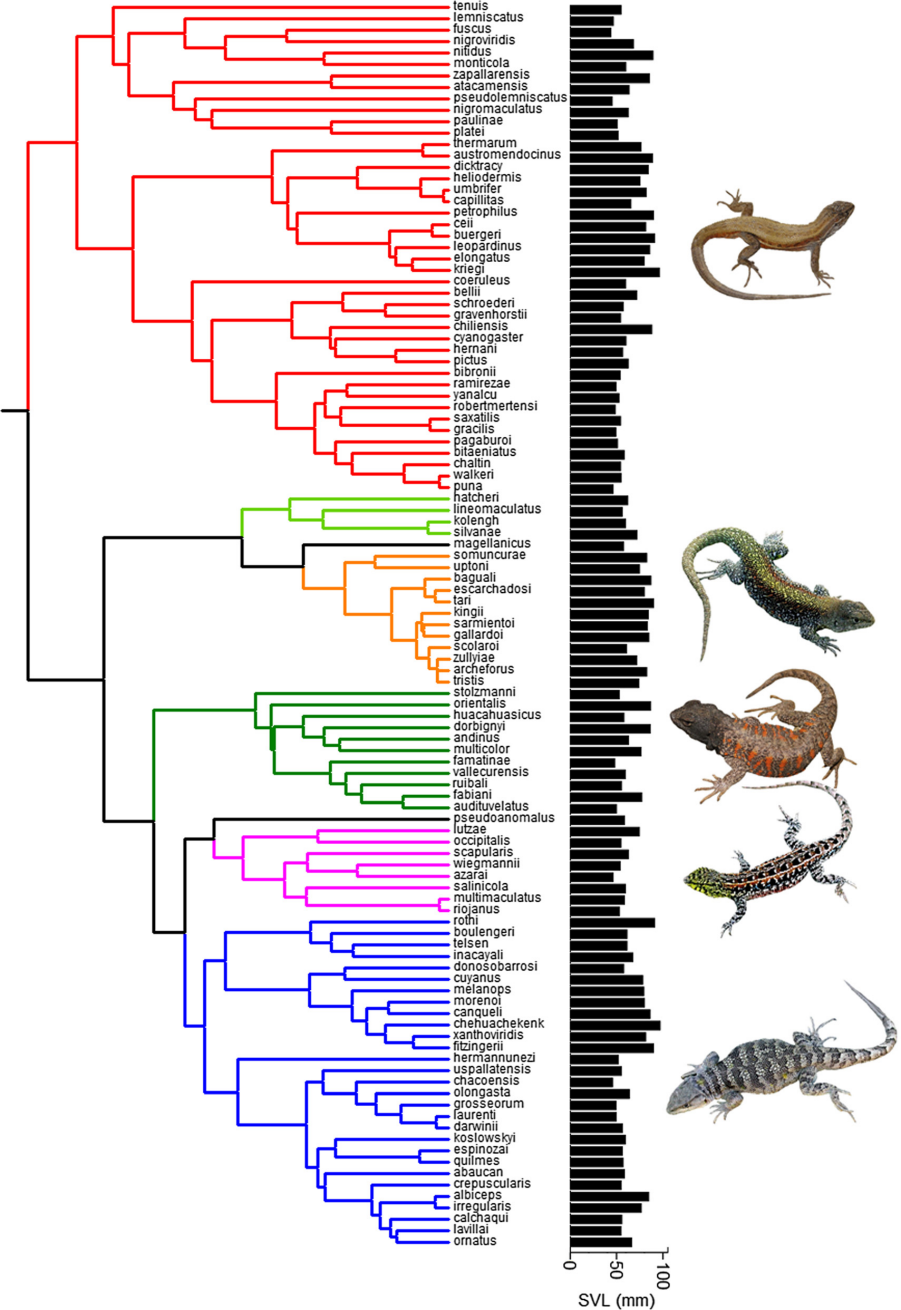
Phylogenetic relationships within the *Liolaemus* radiation showing variation in body size (snout-vent length obtained by averaging male and female SVLs) across species (black bars, in mm). Clade colours indicate the eight main groups (or subgenera) within the genus

Our analyses are based on a multi-gene molecular, time-calibrated phylogenetic tree (Fig. 1), including 109 of the ~240 known *Liolaemus* species (the total number of species is difficult to determine given taxonomic controversies and the lack of reliable diagnoses for several species), extracted from Pyron *et al.*’s [37] comprehensive tree of squamates. The tree was time-calibrated using recent estimates obtained from molecular phylogenies of the major clades within *Liolaemus* [26], and based on the genus’ fossil record [38–40]. We set the origin of the *Liolaemus* crown group radiation (beginning with the latest common ancestry between the subgenera *Eulaemus* and *Liolaemus sensu stricto*) at 19.25 million years ago (Mya). This time represents the average between paleontological and molecular estimates, which place the origin of the crown group radiation, respectively, at 18.5 and 20 Mya.

### Analyses of lineage diversification

Analyses based on the time-calibrated phylogenetic tree were performed to quantify the evolutionary tempo and mode of diversification in *Liolaemus*, with focus on both lineage and body size diversity. To quantify historical rates of species accumulations (i.e., tests of the prediction that diversification has slowed down over time following an early burst) we created a lineage through-time (LTT) plot implemented in the R package ‘ape’ [41]. For the LTT curve, we first implemented Pybus & Harvey’s [42] Monte Carlo Constant Rate (MCCR) test. This analysis calculates the *γ* statistic for incompletely sampled phylogenies, by comparing the distribution of inter-node distances between the tree root and its temporal midpoint to the distribution of distances between the temporal midpoint and the tree tips [43]. Negative *γ* values indicate that inter-node distances between the root and the midpoint are shorter than the distances between the midpoint and the tips, and hence, that most branching events occurred earlier in the evolutionary history of the clade, a pattern consistent with a decline in the rate of species accumulation over time (i.e., an ‘early burst’ model of diversification). When lineage diversification follows a constant rate process, the branching events are evenly distributed throughout the tree, with *γ* being normally distributed and with a mean of 0. Given that incomplete taxon sampling in a phylogeny increases type I error rates in diversification analyses, the MCCR test computes corrected *γ* distributions through simulations of phylogenies to the known clade size (~240 species in *Liolaemus*) under the null hypothesis of a constant rate pure-birth diversification process. Species are then randomly pruned from the simulated trees to replicate incomplete sampling (109 species are included in our tree; see above). Our analysis is based on 10,000 Monte Carlo simulations. The MCCR test was conducted using the ‘laser’ package in R [44].

We then analysed the diversification dynamics that are more likely to have shaped the LTT trend of *Liolaemus* species accumulation by fitting multiple evolutionary models that rely on different evolutionary processes. We used Etienne et al.’s [45] maximum-likelihood fitting-model approach to test four alternative hypotheses of diversification. This technique employs a hidden Markov model (HMM) approach to calculate the likelihood of a phylogenetic history under multiple diversity-dependent birth-death models of diversification. These models account for the influence that species other than those included in the phylogeny (i.e., both extinct species and species missing from the phylogeny) may have on historical rates of diversification (given that speciation rates are a function of existing species at each point in time). Therefore, this approach is comparable to the results produced by the MCCR test above as both techniques consider the potential effects of species missing from the tree [43]. Two of the four fitted models assume constant diversification rates. These are the pure-Birth (or Yule) model, which assumes no extinctions, and the constant rate birth-death model (crBD), which allows extinctions but assumes that the rates of speciation and extinction remain constant through time and across lineages. The other two models, density-dependent logistic (DDL + E) and density-dependent exponential (DDE + E), assume diversity-dependence and thus quantify diversification rates as functions of changes in accumulating diversity over time (while accounting for extinctions, E). While the DDL + E models linear rate changes in diversification, the DDE + E models exponentially declining speciation rates as a function of extant lineage diversity at any point in time. We fitted all four models under two alternative assumptions about the proportion of missing species in the phylogeny. First, we assumed that the *Liolaemus* clade consists of its currently known 240 species. We then assumed that the genus consists of many more species than those currently reported, and that our phylogeny only accounts for 30 % of the ‘real’ diversity of the lineage. For both scenarios, we fitted all four models using the R package ‘DDD’ [45].

To evaluate the best-fit model, we employed the Akaike Information Criterion (AIC) approach [46]. We report the bias-corrected version of AIC, referred to as AICc [47, 48]. The goodness of fit of candidate evolutionary models is determined by identifying the lowest AICc scores, and hence, when shown as ΔAICc scores (the difference between the best or lowest AICc, and the AICc of each alternative model), then the best model has ΔAICc = 0 [47, 48].

### Body size data

To evaluate the potential relationship between clade diversification and phenotypic evolution during the radiation of *Liolaemus*, we investigated the rates and trajectories of body size diversification. We focus on body size as it is the single most important morphological trait that influences the majority of ecological and evolutionary processes via its correlation with most components of organismal form and function [49, 50]. In addition, body size is often considered to be a key morphological indicator of niche in natural populations [49, 51]. Also, in *Liolaemus* in particular, body size is ideally suited for diversification analyses as existing evidence suggests that its variation is not predictably influenced by geographic/climatic clines [28, 30, 34], it varies with numbers of coexisting species (Pincheira-Donoso, unpublished observation), and other phenotypic traits observed to respond to ecological pressures in other lineages (e.g., body proportions, [1]) vary in rather unpredictable ways when linked to, for example, habitat characteristics [30, 52, 53]. We used snout-vent length (SVL), the traditional proxy for body size in lizards [54–56]. For the analyses, we collated an extensive body size dataset (Additional file 1) consisting of 6,500+ adult individuals (adulthood was estimated based on body sizes reported in previous studies, [30–32, 34]), representing >85 % of the currently known species diversity within the genus. To obtain SVL for each species, we averaged male and female SVL values, calculated independently using the upper two-thirds of the size range available for each sex in each species [30, 57]. Although maximum SVL has been extensively used as a proxy for size in lizards, it has been shown that the use of extreme values may result in body size overestimations [58]. In contrast, the use of intermediate percentiles between the maximum recorded value and the mean from the entire adult sample provides accurate estimates of asymptotic size [58]. The entire dataset was collected by the same person (DPD) to control for error arising from inter-individual measurements (e.g., [57]). The species included in our dataset encompass the entire phylogenetic, phenotypic, ecological, and geographic diversity known in *Liolaemus* [30, 52, 53], and therefore, they provide an adequate sample of the body size diversity in this genus (Fig. 1).

### Modelling body size evolution

We investigated the evolutionary dynamics of body size throughout the phylogenetic history of *Liolaemus* using two quantitative approaches based on our time-calibrated phylogeny. First, we quantified the tempo and mode of body size diversification by fitting four alternative models that describe different evolutionary dynamics: the Brownian-motion model (BM, which describes a random walk of trait evolution along branches in the phylogeny, with increase in trait variance centered around the initial value at the root of the tree, and increasing with the distance from the tree root; [59]), the Ornstein-Uhlenbeck model (OU, which assumes that once traits have adaptively evolved, stabilizing selection pulls the trait values around an adaptive optimum for the trait; [60]), the Early-Burst or “niche-filling” model (EB, which describes exponentially increasing or decreasing rates of evolution over time based on the assumption that niches are saturated by accumulating species within a lineage; [8]), and the Delta model (a time-dependent model of trait evolution, which describes the effects that early versus late evolution in the tree have on the rates of trait evolution; it returns a *δ* value which indicates whether recent evolution has been fast when *δ* > 1, or slow when *δ* < 1; [61]). Comparisons of goodness of fit for these models were performed through the Akaike Information Criterion (AIC) [46]. Selection of the best evolutionary model is based on the same AICc approach described above for model-selection of lineage accumulation. Model implementation and fitting was conducted with the R package ‘geiger’ [62]. We then investigated whether the distribution of body size in *Liolaemus* has evolved around a given number of SVL optima (i.e., whether stabilizing selection has promoted macroevolutionary convergences of the trait against one or more such peaks), using the ‘surface’ package in R [63, 64]. This surface method fits an adaptive radiation model in which lineages on a phylogeny may experience convergent shifts towards adaptive optima on a macroevolutionary Simpsonian landscape, importantly, without assumptions of whether some lineages correspond to particular optima [63, 64]. Based on an OU model [60] in which all species are pulled against a single adaptive optimum in morphospace, SURFACE employs a stepwise model selection approach based on AIC_c_, which allows for identification of the best model and the numbers and positions of adaptive peaks (i.e., trait ‘regimes’), and hence, for convergence towards these optima over evolutionary time [63, 64].

We then modelled body size disparity through time (DTT). Based on size data from extant species (see above), this approach calculates the mean disparity for the trait over time, and compares the observed body size disparity with that expected under a null model of Brownian-motion by simulating body size evolution 10,000 times across the tree [12]. Then, the average body size disparity obtained from the real and the simulated data are plotted against node age to calculate the morphological disparity index (MDI). This index quantifies the overall difference in relative disparity for the trait among and within subclades (i.e., differences in the range of variation) compared with the expectation under the null Brownian motion model [13, 62, 65]. Negative MDI values indicate lower than expected trait relative disparity under Brownian motion (i.e., low average subclade relative disparity), which in turn indicates that most disparity occurs among subclades, and therefore, that they occupy smaller and more isolated areas of the morphospace [12]. In contrast, positive MDI values indicate that relative disparity among subclades shows stronger overlap in morphospace occupation [12]. Trait disparity analyses were conducted using the R package ‘geiger’ [62]. The plot projecting the *Liolaemus* phylogeny onto the body size morphospace (against time since the root), based on ancestral node estimations using maximum likelihood [66] is shown in Fig. 3 (see legend for details), and was built using the R package ‘phytools’ [67].

We finally investigated the influence of body size on macroevolutionary lineage diversification in *Liolaemus*. We employed the phylogenetic likelihood-based approach Quantitative State Speciation and Extinction (QuaSSE) implemented in the R package ‘diversitree’ [68]. This method fits evolutionary models based on the distribution of extant characters (body size) on a phylogeny, under the assumption that diversification follows a birth-death process and that a species can be characterized by its mean value of the measured trait, which affects diversification through its effect on the speciation-extinction rates (where rate of speciation is *λ*, and the rate of extinction is *μ*, see [69]). Evolutionary models are fitted by adding a ‘drift’ or ‘directional’ parameter (*φ*), which describes the deterministic (or directional) component of character evolution. That is, the expected rate of character change over time as a function of selection or other process which determines a directional tendency [68, 70]. Thus, this term does not refer to genetic drift specifically. After adding the drift term, the likelihood functions created by QuaSSE describe diversification by a constant, linear, sigmoidal, or hump-shaped function of log body size [68]. Identification of the best evolutionary model is performed via the AIC approach (see above).

## Results

### Diversification rates and evolutionary models

**Fig. 2:**
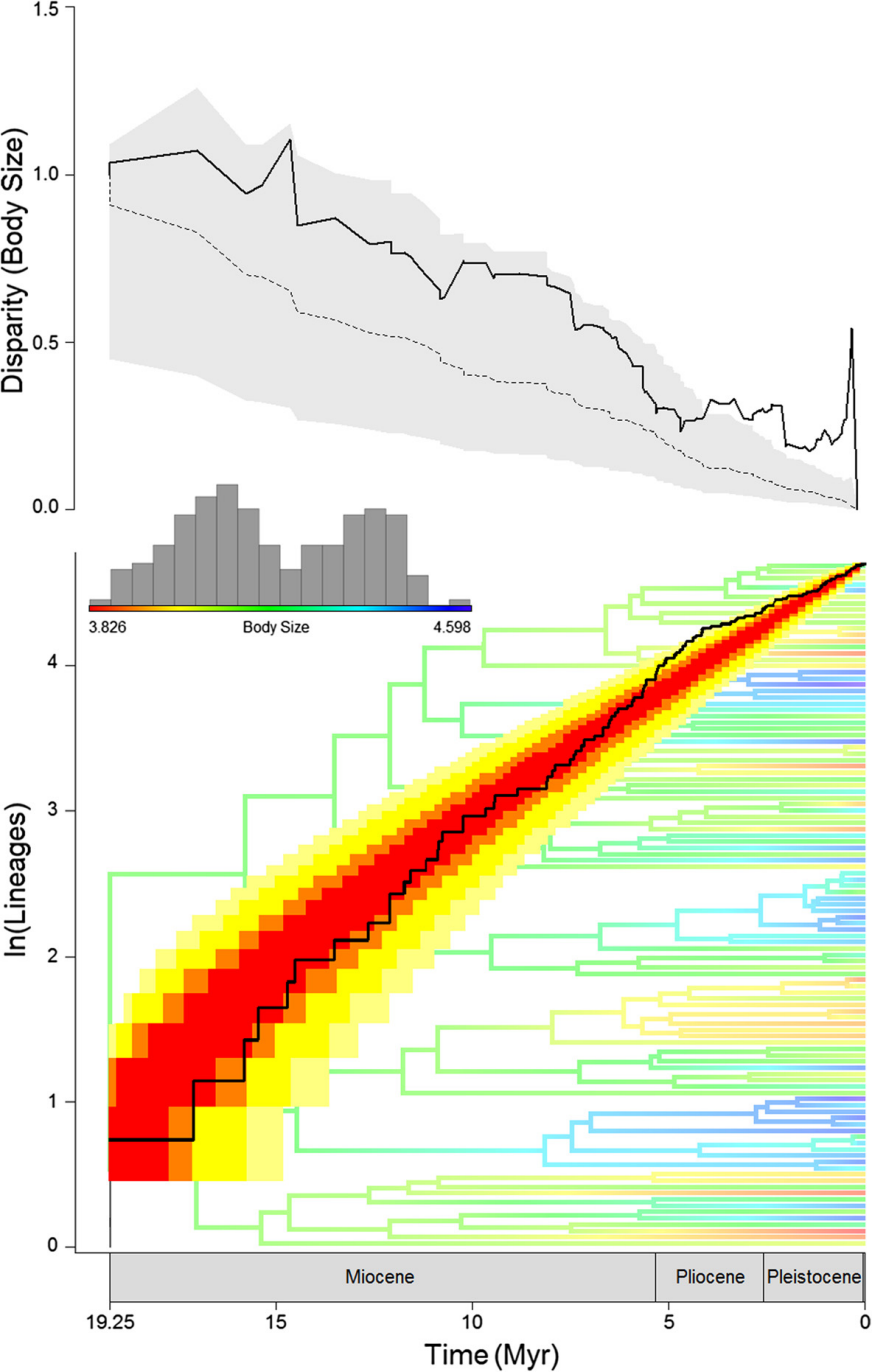
Tempo and mode of macroevolutionary diversification in *Liolaemus* lizards. The bottom plot shows the lineage through time (LTT) curve of species accumulation over time (solid line) and the 95 % (yellow area) to 50 % (red area) confidence intervals (note the most recent pulse is borderline). The phylogenetic tree in the background shows a maximum-likelihood phylogenetic reconstruction of ancestral body sizes (ln-transformed) along the branches and nodes of the tree, and the interspecific range is shown in the coloured bar with the frequency distribution of SVL of the entire genus. The top plot shows mean subclade disparity through time (DTT) for body size (solid line), compared with the median subclade DTT (calculated based on 10,000 simulations) of phenotypic evolution on the genus phylogeny under a Brownian motion model (dashed line). The grey shaded area represents the 95 % confidence interval of DTT range based on simulations of body size disparity

The results from the MCCR analysis, as shown by the lineage through-time plot (Fig. 2), suggest that lineage accumulation over time in *Liolaemus* differs from the pattern expected under the null pure-birth model of constant rate diversification (*γ* = −3.84), but not significantly so (*P* = 0.13). Although the shape of the LTT plot is not consistent with a traditional early-burst curve of diversification, two slight pulses of increased diversification rates followed by declines can be observed (however, both are contained within the 95 % confidence interval). One of these increases occurs approximately between 12–9 Mya, followed by a slight decline ~8-7 Mya. A subsequent slight increase occurs ~5-4 Mya followed by a decline in the most recent phase of the clade’s history (Fig. 2).

The maximum-likelihood analyses of lineage diversification based on four candidate models identify the diversity-dependent linear model (DDL + E) as the best description of the estimated pattern of evolutionary diversification of *Liolaemus* (Table 1). This finding remains supported when the same models are fitted under the assumption that only 30 % of the ‘real’ diversity of the genus is sampled in the phylogeny, and therefore, these observations are unlikely to be an artefact associated with numbers of known and missing species. However, it is important to note that the ΔAICc values between the DDL + E and the Yule models are small – for the scenario based on the actual numbers of species known and sampled, the difference (1.89, Table 1) is close to the threshold value of 2, which identifies well-supported models. The difference is much smaller (0.68, Table 1) for the scenario that assumes 30 % of the real diversity, which indicates that both models are qualitatively similar. An alternative model-fitting analysis based on the same four models, but using the package ‘laser’ [44], revealed identical results: the DDL model provides the best approximation to the observed pattern of species accumulation over time (results not shown).

**Table 1:** Rates of species accumulation during *Liolaemus* diversification history based on multiple evolutionary models. Fitted models are pure-birth (Yule), birth-death (crBD), density-dependent logistic (DDL + E) and density-dependent exponential diversification (DDE + E). Best-fit of models based on (delta) bias-corrected Akaike Information Criteria (AICc)

Model	*λ*	*μ*	LogL	AICc	ΔAICc
Known missing					
Yule	4.02	0	21.16	−40.28	1.89
crBD	4.02	1.3e-13	21.16	−38.20	3.96
DDL + E	7.09	1.05	24.19	−42.16	0
DDE + E	2.24	0.37	−5.95	18.12	60.29
70 % missing					
Yule	4.20	0	21.64	−41.24	0.68
crBD	4.20	2.5e-14	21.64	−39.16	2.76
DDL + E	7.32	1.25	24.07	−41.92	0
DDE + E	2.24	0.37	−11.18	28.58	70.50

### Tempo and mode of body size evolution

**Fig. 3:**
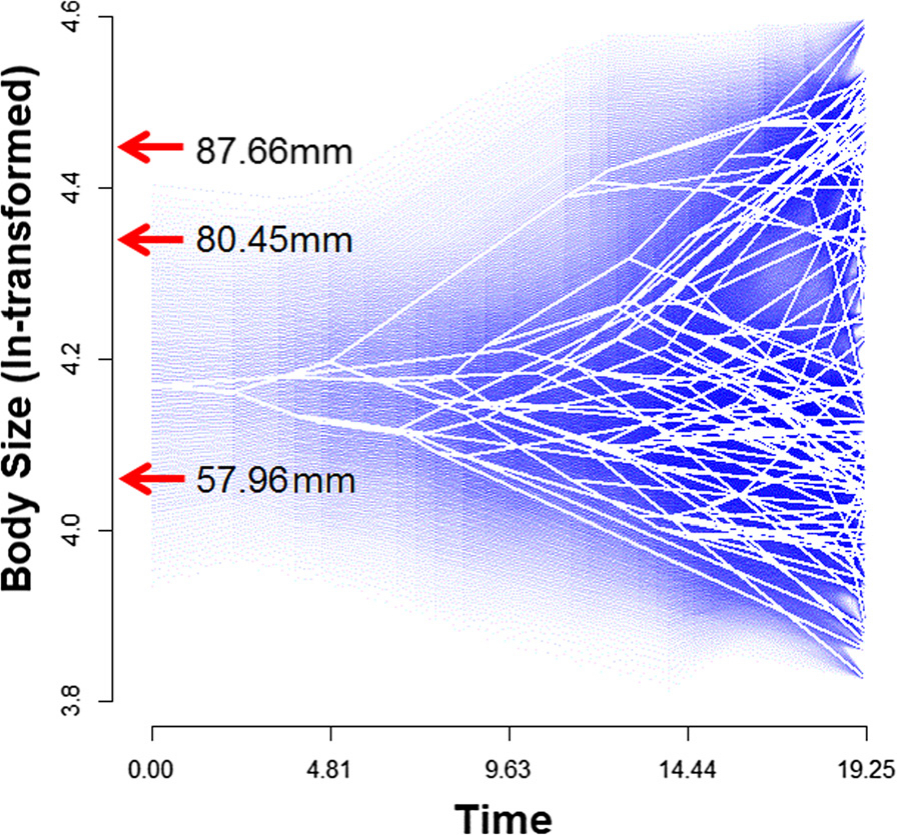
Projection of the *Liolaemus* phylogeny into a morphospace defined by body size (ln-transformed, on *y*) and time since the clade’s origin (on *x*, in My elapsed since the root). Ancestral body size states are estimated using likelihood. The degree of uncertainty is indicated by increasing transparency of the plotted blue lines around the point estimates with the entire range showing the 95 % confidence interval. Red arrows indicate the position of the three body size peaks (in mm) identified by the surface analysis (see text for details)

The analysis of phenotypic DTT reveals that rates of subclade-level diversification in *Liolaemus* body size are consistently higher (positive) than expected under a Brownian motion model of evolution (MDI = 0.23; Fig. 2). Therefore, *Liolaemus* subclades have extensively diversified in body size and converged to occupy overlapping regions of the lineage’s morphospace (Fig. 3). The DTT plot shows an overall tendency for relative disparity in body size to decrease over time, although there are multiple pulses of increases in diversification. There are two slight pulses during the Miocene (both within the 95 % CI calculated from simulations of body size disparity), one between approximately 15.5-14 Mya, and one between approximately 10.5-7.5 Mya. More notably, however, a strong increase is observed during the Pliocene, in which the trend of body size relative disparity exceeds the 95 % DTT range of the simulated data (Fig. 2). Such high positive relative disparity remains through to the Pleistocene, when the model recovers an unusually high relative disparity peak between approximately 0.6-0.3 Mya (Fig. 2).

Our model-based analyses of body size diversification identified OU as the best approximation to the observed pattern of evolution of this trait in *Liolaemus* (Table 2). Therefore, our results suggest that body size diversification has been subject to stabilizing selection that has forced the expression of the trait around certain adaptive optima over time. The Delta and BM models were, respectively, the next best-fitted models, while the EB model was identified as the weakest approximation to describe the pattern of body size evolution (Table 2). Our subsequent convergence analyses of multiple body size peaks on a Simpsonian landscape revealed that three optima (or body size regimes) exist within *Liolaemus*, suggesting that species are pulled by stabilizing selection around the size optima 57.96 mm, 80.45 mm, and 87.66 mm (Additional file 2: Figures S1 and S2).

**Table 2:** Rates and modes of evolutionary diversification in *Liolaemus* body size based on comparisons of the fit of four evolutionary models. Fitted models are Brownian-motion (BM), Ornstein-Uhlenbeck (OU), Early-Burst (EB) and Delta. Best-fit of models based on (delta) bias-corrected Akaike Information Criteria (AICc)

Model	Model Parameters	*β*	LogL	AICc	ΔAICc
BM	–	0.006	29.74	−55.37	16.12
OU	*α* = 0.122	0.009	38.86	−71.49	0
EB	*a* = −1.0e-06	0.006	29.74	−53.25	18.24
Delta	*δ* = 2.99	0.002	37.48	−68.73	2.76

Finally, our analysis of trait-dependent macroevolutionary diversification identified a negative linear function as the best model. That is, diversification rates increase as a linear function of decreasing body size (Table 3).

**Table 3:** QuaSSE trait-dependent lineage diversification in *Liolaemus*. Analyses based on selection from multiple models described by a linear, sigmoidal or hump-shaped function with (drift) and without a ‘drift’ or directional term added to the model fitting (see text for details). Best-fit of models based on (delta) bias-corrected Akaike Information Criteria (AICc)

Model	LogL	*Χ* ^2^	*P*	AICc	ΔAICc
Linear	−275.11	4.51	0.03	558.61	12.39
Sigmoidal	−275.70	3.34	0.34	564.22	18.00
Hum-shaped	−275.83	3.08	0.38	564.47	18.25
Linear (drift)	−267.82	19.09	7.1e-05	546.22	0
Sigmoidal (drift)	−271.14	12.45	0.01	557.39	11.18
Hum-shaped (drift)	−267.34	20.05	<0.001	549.79	3.58

## Discussion

Rapid early bursts of phylogenetic, phenotypic and ecological diversification within a lineage entering a novel adaptive zone are central components of the definition of a process of adaptive radiation [2, 71]. Our analyses investigating the tempo and mode of evolutionary diversification of one of Earth’s most prolific vertebrate radiations (*Liolaemus* lizards) reveals a density-dependent pattern of lineage accumulation over time (Fig. 2), while in contrast, the evolution of body size does not follow a traditional pattern of adaptive radiation mode of diversification (i.e., it does not conform to an early-burst trajectory). This latter finding is further confirmed by the strong subclade overlap in morphospace revealed by the DTT analysis (Figs. 2 and 3). Instead, body size evolution is best explained by a model based on stabilizing selection (i.e., OU) that pulls the trait towards three convergent adaptive optima during the lineage’s evolutionary history. These multiple species-level size peaks are confirmed by our maximum-likelihood phylogenetic reconstruction of *Liolaemus* ancestral body sizes (Fig. 2). Interestingly, analyses of trait-dependent diversification showed that higher rates of lineage accumulation are associated with smaller body size (Table 3). Traditionally, analyses of both lineage and phenotypic evolution have been employed to address the role of early ecological opportunity followed by density-dependent declines in diversification via niche saturation over evolutionary time (often within the context of island versus continental radiations) [2, 9]. In turn, these phenomena are central to the definition of adaptive radiation [1, 2, 7, 10], and hence, have served to identify lineages that have followed this route of diversification.

### Diversification dynamics and continental evolutionary radiations

Evolutionary diversifications in island systems (e.g., oceanic islands, mainland ‘lake archipelagos’) and in continental settings are widely thought to proceed under different ecological dynamics, and scenarios leading to adaptive radiations are thought to prevail on islands. Indeed, most emblematic examples of adaptive radiations have diversified on island systems [1, 72–74], and the outcomes of evolutionary radiations often differ between island and continental phylogenetically related lineages [22, 75]. High ecological opportunity emerging from lower interspecific competition and high resource abundance are broadly believed to be the basis to trigger adaptive radiations on islands. In contrast, mainlands offer much more complex and competitive environments [1, 21–23]. Therefore, it has been suggested that the ecological opportunity that promotes adaptive radiations on islands may not generally occur in continental systems [e.g., 23], which would explain their differences in radiation patterns.

Ecological opportunity is, however, unlikely to be a feature of islands only. Instead, we argue that ecological opportunity is temporally episodic and dependent on the environmental (i.e., ecological, geographic, climatic) stability of a landmass. Islands are in general more unstable [76], while continents (given their larger area) are more stable over time. Therefore, the emergence of ecological opportunity is more likely to be a function of landmass area, and hence, it may only be less frequent in continents. In fact, continents are known to have been scenarios for active adaptive radiations driven by emergence of ecological opportunity, for example, following mass extinctions [1–3, 72]. In line with these views, the prolific continental radiation of *Liolaemus* lizards has been suggested to be importantly explained by large-scale ecological opportunity [6, 35]. Adaptive radiations can be triggered by extrinsic factors such as the arise of new ecological opportunity via emergence of novel environments, and/or by intrinsic factors (‘key adaptive innovations’) that increase the availability of niches to a diversifying lineage [1–3, 7, 9]. Pincheira-Donoso et al. [6] suggested that the onset of this lizard radiation resulted from a combination of both scenarios. Extrinsically, the emergence and uplift of the Andes over the last ~25My [77, 78] created unprecedented novel ecological opportunity (an enormous new high-elevation ecosystem), which is known to have also promoted biodiversity proliferations in a variety of other organisms [79–82]. Intrinsically, given that low-temperature environments impose strong selection against reptile developing eggs in nests [83, 84], successful colonization of cold Andean climates demanded the evolution of prolonged embryo retention, i.e., viviparity [6]. In support of this view, the overwhelming majority of cold-climate *Liolaemus* species are viviparous [6, 36, 85], and the multiple independent events of phylogenetic oviparity-to-viviparity transitions are strongly correlated with multiple independent invasions of colder environments during the lineage history [6]. Invasions of cold-climate Patagonia have followed exactly the same patterns, thus reinforcing the ‘key innovation’ nature of viviparity [6]. Remarkably, over 55 % of the *Liolaemus* species for which parity mode is known are viviparous [6, 85]. Therefore, this relatively young continental lineage is likely to have adaptively radiated driven by ecological opportunity, and about half of its exceptional diversity potentially evolved as a result of the viviparity innovation that allowed access to exploit such opportunities [6]. Interestingly, as indicated by Schulte et al. [36], our results also suggest that the enormous climatic crises caused by the Pleistocene do not seem to have had an important role in the diversification of *Liolaemus* lineages (Fig. 2).

Our lineage through-time analyses support a density-dependent model of adaptive radiation, as shown by the DDL + E model identified as the best approximation for the diversification within *Liolaemus*. This analysis suggests that *Liolaemus* diversification has tended to decline over time as a function of accumulating species, although both the exponential and the decline phases of the diversification curve are only slightly pronounced (in fact, the Yule model was identified as the next best alternative; Fig. 2). These findings contrast with the model-based analysis of body size diversification, which identified the ‘early burst’ (EB) model as the less preferred alternative (and the OU as the best one), while the relative disparity through-time analysis returned a positive MDI value (i.e., extensive trait diversification, but strong subclade overlap in the morphospace, [12]). Traditionally, *negative* MDI values are interpreted as consistent with phenotypic diversification during adaptive radiation [12, 13, 16, 86]. Therefore, in our analyses, the rates and trajectories of diversification are not consistent between lineage and phenotypic evolution (although a negative relationship between the two seems to have dominated the radiation history of this clade; Table 3). However, we argue that the high phenotypic diversification and morphospace overlap found in *Liolaemus* can in fact be consistent with a process of adaptive radiation. In this lineage, geographic overlap among main subclades tends to be limited [31, 36, 87], compared to other reptile radiations. Therefore, overlap in morphospace does not translate into spatial (i.e., ecological) overlap, and hence, such high phenotypic relative disparity is likely to have evolved independently among subclades in different areas [12]. In other words, the *Liolaemus* genus as a whole may be a collection of replicated and independently radiating subclades where events of diversification are marginally or not influenced by other subclades within the genus. The role for limited spatial overlap among diversifying lineages in the rates of phenotypic diversification has also been suggested elsewhere [2, 8, 12]. The principle is that EB-like diversification is more likely to occur in lineages with a large proportion of sympatric species early in their history, given that saturation of ecological space is directly mediated by species interactions (e.g., competition) and coadaptation [2, 8]. As indicated above, *Liolaemus* subclades tend to ‘specialize’ in different geographic zones, and important part of this territory is a highly complex Andean topography, that further increases spatial isolation between groups of species within subclades [35]. An interesting implication of this phylogeographic pattern is that continental radiations can face opportunities for more complex macroevolutionary patterns to emerge. For example, non-adaptively radiating subclades may evolve within a lineage that is fundamentally an adaptive radiation. Cases like this may also exist in Andean *Liolaemus* subclades, in which sets of morphologically and ecologically similar species occur in isolation from each other along ‘mountain chains’ that run latitudinally. This idea was suggested by Pincheira-Donoso & Nuñez [31] who noted thatsome phenotypically and ecologically similar *Liolaemus* species (e.g., their ‘*nigroviridis*’ group) replace each other along a latitudinal chain of high Andean areas. The same is true for the *Liolaemus*’ sister genus *Phymaturus*, which has emerged as a candidate case of non-adaptive radiation given the same pattern [88, 89].

### What does define an adaptive radiation?

Although our study reveals an apparent disconnection between dynamics of clade and phenotypic diversification, both findings are consistent with evolutionary patterns observed in a diversity of animal lineages [8, 16]. Most notably, Harmon et al. [8] recently showed that the early-bursts of phenotypic diversification traditionally predicted by theory [2, 10] are only rarely observed across numerous cases of adaptive radiations ranging from taxonomically small to large lineages. Therefore, this EB pattern of evolutionary diversification traditionally implied as a central condition to define adaptive radiations is not compatible with the evolutionary history of multiple classic lineages that have been instrumental in shaping the theory of adaptive radiation itself. These replicated findings raise the question of what features define an adaptive radiation.

On one hand, we agree with previous authors [1, 8, 90] that the timing of adaptive radiation is not a necessary condition to define the process. Instead, it is a feature that should be empirically tested to better understand the contexts of diversification of specific clades [1]. In addition, inference of diversification dynamics as functions of ecological processes (e.g., density-dependent diversification via niche saturation over time) from LTT curves can be inaccurate. For instance, while asymptotic functions may not necessarily reflect density-dependent diversification [91–93], failure to identify diversification slowdowns does not rule out a density-dependent diversification [9]. Also, the traditional link between asymptotic diversification patterns and adaptive radiations implicitly assumes that the environments occupied by radiating clades are rather static over their evolutionary histories (i.e., ecological opportunity gets saturated early on, then, adaptive diversification slows down). However, episodes of ecological opportunity can emerge multiple times during the evolutionary history of a lineage (see above). For example, the temporally and spatially spread pulses of Andean uplifts are likely to have generated episodic ecological opportunity during the history of *Liolaemus*, potentially eroding a more pronounced overall asymptotic diversity-dependent curve for the genus (which may at least in part be linked to the, although non-significant, slight pulses of diversification observed in the LTT analysis; Fig. 2). Unquestionably, diversification mediated by niche filling following emergence of ecological opportunity is a central component of processes of adaptive radiation. Therefore, we argue that adaptively radiating lineages are likely to experience bursts of species and phenotypic diversification during their evolutionary history. Yet, as suggested above, these bursts can occur in multiple episodes which are, in turn, likely to be a function of changes in the environment, thus eroding the density-dependent signature of diversification. Consequently, we support the view [1, 3, 90] that an adaptive radiation should be defined as the diversification of a single lineage into a variety of species adapted to exploit different portions of the multidimensional spectrum of ecological resources driven by divergent natural selection. On the other hand, as indicated above, we suggest that interpretations of the signals of diversification mode inferred from relative disparity through-time analyses should be based on multiple factors, rather than on the extent of subclade overlap in morphospace (i.e., the MDI value) alone.

## Conclusions

Our study reveals that lineage diversification in the continental genus *Liolaemus*, one of Earth’s most prolific vertebrate radiations, conforms to a diversity-dependent model. This process is traditionally interpreted as adaptive radiation through niche filling [2, 8, 94]. Consistent with continuous large-scale environmental changes and emergence of ecological opportunity caused by the uplift of the Andes, this radiation shows some slight signals of episodic pulses of lineage accumulation. Therefore, and regardless of whether these pulses are linked to episodic ecological opportunity offered by the Andes, we suggest that ecological opportunity in continents can be strong and a crucial driver of adaptive radiations, but may emerge less frequently compared to islands. Body size diversification, in contrast, does not follow a niche filling process (it conforms to a multiple-peak OU model and shows a positive MDI value). We argue that depending on factors such as the nature (whether it is episodic, spatially spread) of ecological opportunity, lineage distribution, and the functional contribution of traits to adaptive diversification, models of diversification between lineages and phenotypes can differ. Finally, in agreement with previous authors [1, 8, 90], we suggest that adaptive radiations should not be defined solely based on evidence for early-burst processes. Instead, we advocate a more general definition based on evidence for diversification of an ancestor into multiple species adapted to different ecological niches.

## Availability of supporting data

The body size data set supporting the results of this article is included within the article as Supplementary material 1.

### Research ethics

Research ethical approval and consent are not applicable to this study, since the study involves no human or live animal subjects.

## Additional files

Additional file 1:
**Body size data (snout-vent length, in mm) used for analyses in this study.** List of species names follows the species sequence in the phylogeny (Fig. 1). (DOCX 13 kb) Additional file 2: Figure S1. Surface analysis showing the three inferred adaptive peaks (‘regimes’) of body size (large circles in blue, green and red), and the distribution of species body sizes clustering around these peaks (small circles). **Figure S2.** Surface analysis depicting phylogenetic convergences of body size in Liolaemus. The three colours (blue, green and red) represent the body size peaks shown in the Supplementary Figure S1 above. (DOCX 254 kb)

## Abbreviations

AIC: Akaike Information Criterion
BM: Brownian motion model
crBD: constant rate birth-death model
DDL + E: density-dependent logistic model
DDE + E: density-dependent exponential model
DTT: disparity through time
EB: Early burst model
HMM: hidden Markov model
LTT: lineage through-time plot
MCCR: Monte Carlo Constant Rate
MDI: morphological disparity index
m: metres
My: millions of years
Mya: millions of years ago
OU: Ornstein-Uhlenbeck model
QuaSSE: Quantitative State Speciation and Extinction
SVL: snout-vent length
