# Supplementary material for: What defines an adaptive radiation? Macroevolutionary diversification dynamics of an exceptionally species-rich continental lizard radiation
Source: BMC Evol Biol. 2015 Aug 7;15:153. doi: 10.1186/s12862-015-0435-9 (PMC4527223; doi:10.1186/s12862-015-0435-9)
Supplement: Additional file 1: — Body size data (snout-vent length, in mm) used for analyses in this study. List of species names follows the species sequence in the phylogeny (Fig. 1). (DOCX 13 kb) [file 12862_2015_435_MOESM1_ESM.docx]

**Supplementary Material 1.** Body size data (snout-vent length, in mm) used for analyses in this study. List of species names follows the species sequence in the phylogeny (Fig. 1).

| **Species** | **SVL (mm)** | **ln(SVL)** |
| --- | --- | --- |
| *Liolaemus tenuis*  *Liolaemus lemniscatus*  *Liolaemus fuscus*  *Liolaemus nigroviridis*  *Liolaemus nitidus*  *Liolaemus monticola*  *Liolaemus zapallarensis*  *Liolaemus atacamensis*  *Liolaemus pseudolemniscatus*  *Liolaemus nigromaculatus*  *Liolaemus paulinae*  *Liolaemus platei*  *Liolaemus thermarum*  *Liolaemus austromendocinus*  *Liolaemus dicktracy*  *Liolaemus heliodermis*  *Liolaemus umbrifer*  *Liolaemus capillitas*  *Liolaemus petrophilus*  *Liolaemus ceii*  *Liolaemus buergeri*  *Liolaemus leopardinus*  *Liolaemus elongatus*  *Liolaemus kriegi*  *Liolaemus coeruleus*  *Liolaemus bellii*  *Liolaemus schroederi*  *Liolaemus gravenhorstii*  *Liolaemus chiliensis*  *Liolaemus cyanogaster*  *Liolaemus curicensis*  *Liolaemus pictus*  *Liolaemus bibronii*  *Liolaemus ramirezae*  *Liolaemus yanalcu*  *Liolaemus robertmertensi*  *Liolaemus saxatilis*  *Liolaemus gracilis*  *Liolaemus pagaburoi*  *Liolaemus bitaeniatus*  *Liolaemus chaltin*  *Liolaemus walkeri*  *Liolaemus puna*  *Liolaemus hatcheri*  *Liolaemus lineomaculatus*  *Liolaemus kolengh*  *Liolaemus silvanae*  *Liolaemus magellanicus*  *Liolaemus somuncurae*  *Liolaemus uptoni*  *Liolaemus baguali*  *Liolaemus escarchadosi*  *Liolaemus tari*  *Liolaemus kingii*  *Liolaemus sarmientoi*  *Liolaemus gallardoi*  *Liolaemus scolaroi*  *Liolaemus zullyae*  *Liolaemus archeforus*  *Liolaemus tristis*  *Liolaemus stolzmanni*  *Liolaemus orientalis*  *Liolaemus huacahuasicus*  *Liolaemus dorbignyi*  *Liolaemus andinus*  *Liolaemus multicolor*  *Liolaemus famatinae*  *Liolaemus vallecurensis*  *Liolaemus ruibali*  *Liolaemus fabiani*  *Liolaemus audituvelatus*  *Liolaemus pseudoanomalus*  *Liolaemus lutzae*  *Liolaemus occipitalis*  *Liolaemus scapularis*  *Liolaemus wiegmannii*  *Liolaemus azarai*  *Liolaemus salinicola*  *Liolaemus multimaculatus*  *Liolaemus riojanus*  *Liolaemus rothi*  *Liolaemus boulengeri*  *Liolaemus telsen*  *Liolaemus inacayali*  *Liolaemus donosobarrosi*  *Liolaemus cuyanus*  *Liolaemus melanops*  *Liolaemus morenoi*  *Liolaemus canqueli*  *Liolaemus chehuachekenk*  *Liolaemus xanthoviridis*  *Liolaemus fitzingerii*  *Liolaemus hermannunezi*  *Liolaemus uspallatensis*  *Liolaemus chacoensis*  *Liolaemus olongasta*  *Liolaemus grosseorum*  *Liolaemus laurenti*  *Liolaemus darwinii*  *Liolaemus koslowskyi*  *Liolaemus espinozai*  *Liolaemus quilmes*  *Liolaemus abaucan*  *Liolaemus crepuscularis*  *Liolaemus albiceps*  *Liolaemus irregularis*  *Liolaemus calchaqui*  *Liolaemus lavillai*  *Liolaemus ornatus* | 56.944  48.787  45.877  70.375  91.450  62.243  87.719  65.712  47.450  64.663  52.958  53.814  78.375  91.023  86.300  77.275  84.000  67.700  92.019  83.611  93.224  88.014  81.994  98.394  62.080  73.757  59.247  56.561  90.230  62.384  58.826  64.665  56.023  51.550  54.700  50.850  56.550  51.333  53.150  60.350  56.570  56.965  48.486  64.291  58.279  61.877  74.183  59.745  84.798  76.950  89.246  82.277  92.106  86.461  85.510  86.973  63.077  73.854  84.688  76.120  55.142  89.000  60.060  88.350  64.952  78.383  50.150  61.525  57.516  79.265  52.130  60.833  76.755  56.795  64.818  56.041  48.500  61.807  60.597  55.250  93.317  63.416  63.475  69.708  60.000  80.377  81.454  82.250  88.304  99.250  83.604  92.017  54.035  57.452  47.903  65.970  51.500  51.796  58.532  61.557  58.374  59.069  60.655  56.957  86.900  78.922  57.800  56.890  68.380 | 4.04207  3.88746  3.82598  4.25384  4.51579  4.13104  4.47414  4.18528  3.85967  4.16919  3.96950  3.98553  4.36150  4.51111  4.45782  4.34737  4.43081  4.21508  4.52199  4.42618  4.53501  4.47750  4.40665  4.58898  4.12843  4.30078  4.08172  4.03533  4.50237  4.13332  4.07459  4.16923  4.02577  3.94255  4.00186  3.92888  4.03512  3.93835  3.97311  4.10016  4.03547  4.04245  3.88128  4.16343  4.06525  4.12516  4.30653  4.09009  4.44027  4.34315  4.49139  4.41009  4.52294  4.45970  4.44863  4.46560  4.14436  4.30209  4.43897  4.33231  4.00992  4.48863  4.09534  4.48130  4.17365  4.36160  3.91501  4.11945  4.05206  4.37279  3.95374  4.10813  4.34061  4.03944  4.17158  4.02610  3.88156  4.12402  4.10425  4.01186  4.53601  4.14972  4.15065  4.24432  4.09434  4.38673  4.40004  4.40976  4.48078  4.59764  4.42609  4.52197  3.98963  4.05096  3.86918  4.18920  3.94158  3.94731  4.06959  4.11997  4.06688  4.07871  4.10520  4.04230  4.46475  4.36847  4.05698  4.04113  4.22508 |
