# Supplementary material for: What defines an adaptive radiation? Macroevolutionary diversification dynamics of an exceptionally species-rich continental lizard radiation
Source: BMC Evol Biol. 2015 Aug 7;15:153. doi: 10.1186/s12862-015-0435-9 (PMC4527223; doi:10.1186/s12862-015-0435-9)

**SUPPLEMENTARY MATERIAL 2: Figures**

**What defines an adaptive radiation? Macroevolutionary diversification dynamics of an exceptionally species-rich continental lizard radiation**

Daniel Pincheira-Donoso, Lilly P. Harvey & Marcello Ruta

**Supplementary Figure S1.** Surface analysis showing the three inferred adaptive peaks (‘regimes’) of body size (large circles in blue, green and red), and the distribution of species body sizes clustering around these peaks (small circles).


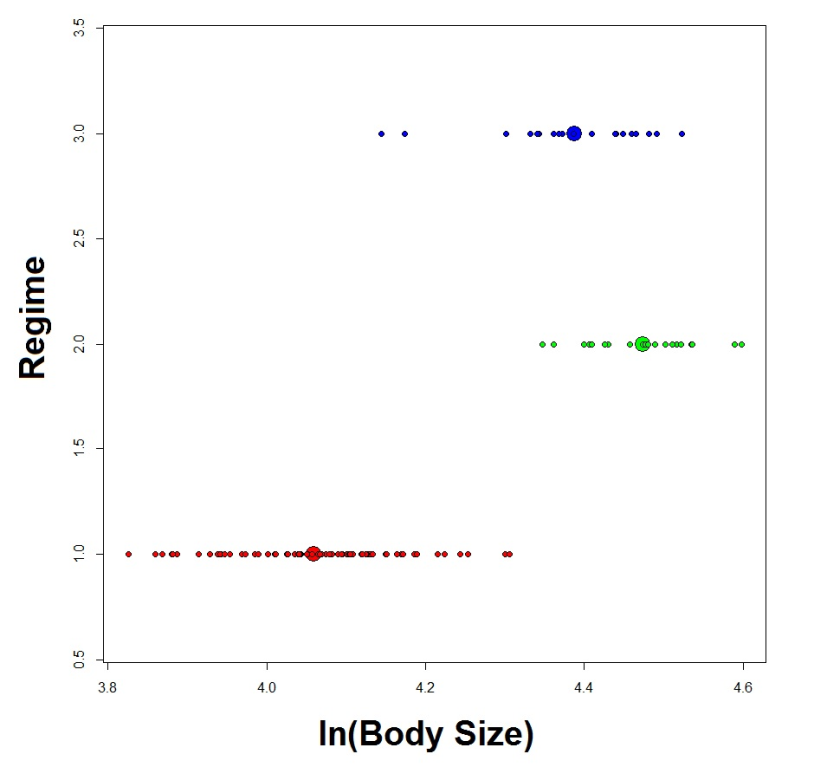


**Supplementary Figure S2.** Surface analysis depicting phylogenetic convergences of body size in Liolaemus. The three colours (blue, green and red) represent the body size peaks shown in the Supplementary Figure S1 above.


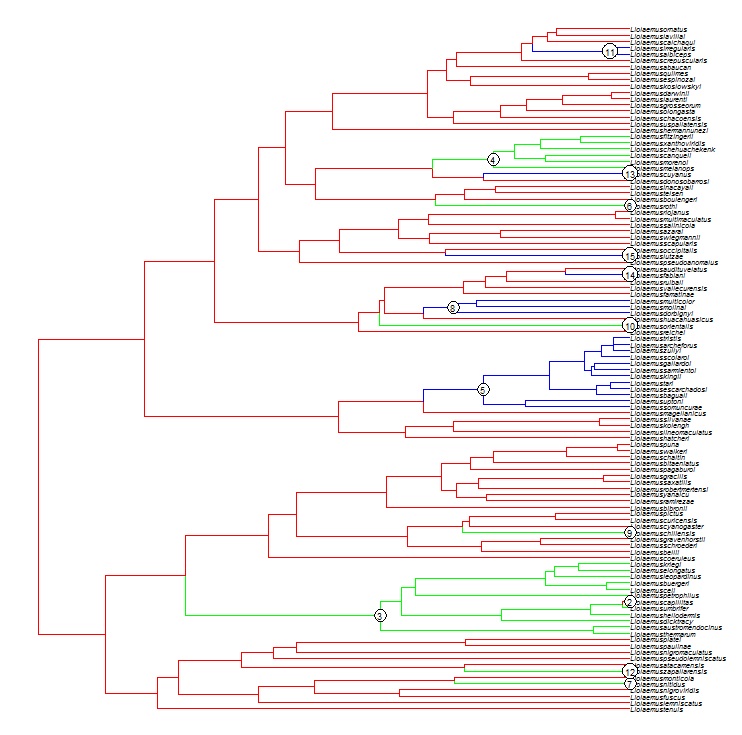

Supplement: Additional file 2: Figure S1. — Surface analysis showing the three inferred adaptive peaks (‘regimes’) of body size (large circles in blue, green and red), and the distribution of species body sizes clustering around these peaks (small circles). Figure S2. Surface analysis depicting phylogenetic convergences of body size in Liolaemus. The three colours (blue, green and red) represent the body size peaks shown in the Supplementary Figure S1 above. (DOCX 254 kb) [file 12862_2015_435_MOESM2_ESM.docx]
